# An Irish experience of robotic adrenalectomy: Transitioning from open to robotic surgery

**DOI:** 10.1007/s11845-026-04428-6

**Published:** 2026-05-26

**Authors:** R. Walsh, J. Walsh, N. Hardy, T. Harding, A. Pathanki, G. McEntee, J. Conneely

**Affiliations:** 1 Royal College of Surgeons of Ireland, 123 St Stephen’s Green, Dublin 2, D02 YN77 Ireland; 2 Department of Hepatobiliary Surgery, The Mater Misericordiae University Hospital, Eccles Street, Dublin 7, Ireland

**Keywords:** Adrenalectomy, Minimally invasive surgery, Perioperative outcomes, Robotic surgery, Surgical transition

## Abstract

**Background:**

Minimally invasive techniques have become the gold-standard for the surgical management of the majority adrenal pathologies. No consensus exists over the benefit of robotic over laparoscopic adrenalectomy. The aim of the study was to analyse the authors’ experience of adrenalectomy with emphasis on its transition to robotic approaches.

**Methods:**

Retrospective data were collated from a prospectively maintained database of all consecutive adult patients undergoing adrenalectomy at the study centre between 1st January 2015 to 1st June 2025.

**Results:**

61 adrenalectomies were included in the study at a median patient age of 54 years (IQR 43—67). 34% of these were for phaeochromocytoma. Open approaches prevailed at the start of the study but over-all, half the cases were performed robotically. Mean operative time reduced over the study period from 264 min in 2015 to 108 min in 2025. Median length of stay (LOS) was 5 days (IQR 4—7).

**Conclusion:**

This case series reflects a change in approach to adrenalectomy. The 10-year period demonstrated a marked transition to robotic adrenalectomy, paralleled by rising case volumes and significant improvements in length of stay, operative time, and blood loss. While the overall improvements are likely multifactorial (learning curve effects, increasing institutional experience, evolving case selection, and rising case volumes)—this study demonstrates the safety and feasibility of robotic adrenalectomy.

## Introduction

Adrenalectomy has evolved significantly since its inception as an open procedure by Sargent in 1912 [1]. The introduction of laparoscopic adrenalectomy by Gagner in 1992 marked a shift toward minimally invasive techniques, offering reduced morbidity and faster recovery [2]. Further advancement came with robotic adrenalectomy, first described by Desai in 2002, leveraging enhanced dexterity, 3D visualization, and precision provided by systems like the Da Vinci Xi [3]. These developments have been supported by substantial evidence demonstrating the safety and efficacy of laparoscopic and robotic approaches over traditional open surgery, particularly for benign and smaller lesions [4–6].

In Ireland, adrenal surgery is performed across various centres, with increasing incidentalomas detected due to advanced imaging [7]. At The Mater Misericordiae University Hospital (MMUH), a tertiary referral centre, the Hepatobiliary and Pancreatic (HPB) service has transitioned from open to minimally invasive methods, incorporating robotic technology. This study retrospectively reviews a 10-year experience (January 2015 to June 2025) to evaluate changes in surgical practice, case volumes, and key perioperative outcomes, providing insights into the practical implementation of robotic adrenalectomy in an Irish context. The findings were presented at the 16th Biennial Congress of the European-African Hepato-Pancreato-Biliary Association (E-AHPBA) 2025 in Dublin, June 10—12.

## Methods

### Study design and population

This was a retrospective cohort study of all adrenalectomies performed by the HPB service at MMUH over a 10-year period from January 2015 to June 2025. Inclusion criteria encompassed all procedures in patients aged > 18 years. Exclusion criteria included combined cases (e.g., adrenalectomy with concurrent major procedures) and paediatric cases. Data was extracted from electronic medical records and the hospital’s surgical database. Given the retrospective observational nature of the study, STROBE reporting guidelines were followed.

### Data collection

Variables collected included patient demographics (age, gender, lesion laterality), histological diagnosis, lesion size, surgical approach (open, laparoscopic, or robotic using Da Vinci Xi), operative time (minutes), estimated blood loss (mL), and length of hospital stay (LOS, days). Annual case volumes and trends in surgical approaches were analysed to assess practice evolution Tables 1 and 2.

**Table 1:** Baseline characteristics

Characteristic	Value
Median age (years)	54
Male/Female	29/32
Laterality (Left/Right/Bilateral/Para-aortic)	23/34/1/3
Approach (Open/Laparoscopic/Robotic)	14/13/34

**Table 2:** Histology and lesion size

Histology	n (%)	Mean Size (cm)
PPGL	20 (33)	4.50
Adenoma/Other benign	24 (39)	3.45
Metastasis	7 (11)	5.50
Primary malignancy	6 (10)	18.33
Myelolipoma	3 (5)	7
Other	1 (2)	3
**Overall**	-	6.27
**Open**	-	9.02
**Minimally Invasive**	-	3.74

PPGL: Pheochromocytoma/paraganglioma

### Statistical analysis

Continuous variables were reported as medians or means as appropriate. Normality was assessed using the Shapiro–Wilk test and homogeneity of variances using Levene’s test. Because data was non-normally distributed, all between-group comparisons of continuous and categorical outcomes were performed with Mann–Whitney U test and chi-square test respectively. Multivariate regression was not performed given small sample size. Trends over time were visualized using line and bar graphs. Statistical significance was set at p < 0.05. Analyses were performed using SPSS version 27.0 (IBM Corp., Armonk, NY). Full ethical approval was received from the MMUH research ethics board 12th March 2025 (Ref: 1/378/2456 TMR).

## Results

A total of 61 adrenalectomies were performed during the study period. Baseline characteristics included a median age of 54 years (IQR 43—67), with 29 males (48%) and 32 females (52%). Lesion laterality was left in 23 cases (38%), right in 34 (56%), bilateral in 1 case (2%), and para-aortic in 3 cases (5%).

### Changes in surgical approaches and case volumes

Surgical approaches evolved markedly over the 10 years. Initially, open procedures dominated, accounting for all cases in 2015 (n = 3) and 2016 (n = 3). Laparoscopic adrenalectomy was introduced in 2017 (n = 2) and peaked in 2018 (n = 3). Robotic adrenalectomy began in 2019 (n = 1) and rapidly increased, becoming the predominant approach by 2021 (n = 3) and reaching highs of 10 cases in 2023, 8 in 2024, and 7 in 2025 (up to June).

Of the 47 minimally invasive cases, there was a single conversion from laparoscopic to open for intra-operative blood loss.

**Fig. 1:**
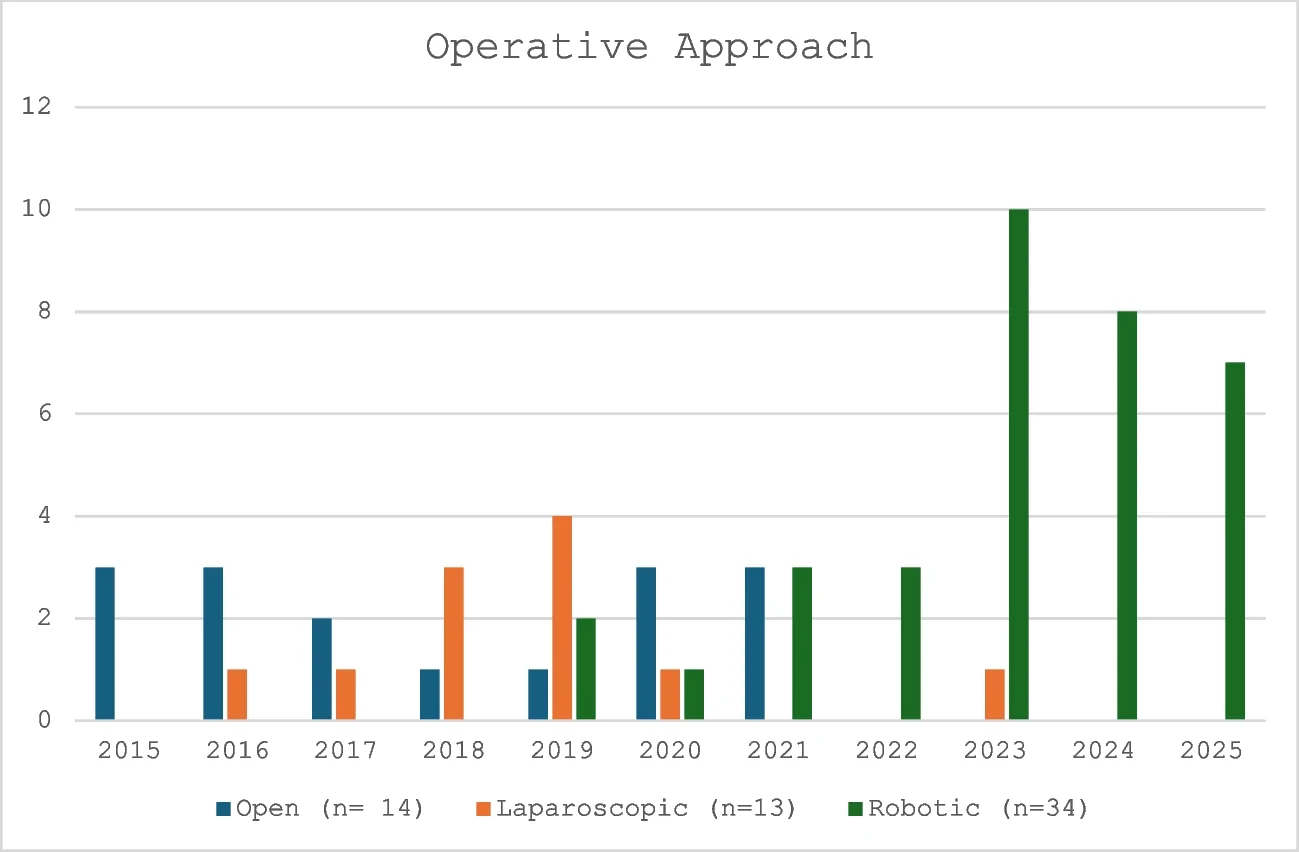
Annual case volume by approach (2015–2025)

Case volumes increased substantially, from 3 per year in 2015 to a peak of 10 in 2023, reflecting service expansion and greater adoption of minimally invasive techniques (Fig. 1).

### Histology and lesion size

**Fig. 2:**
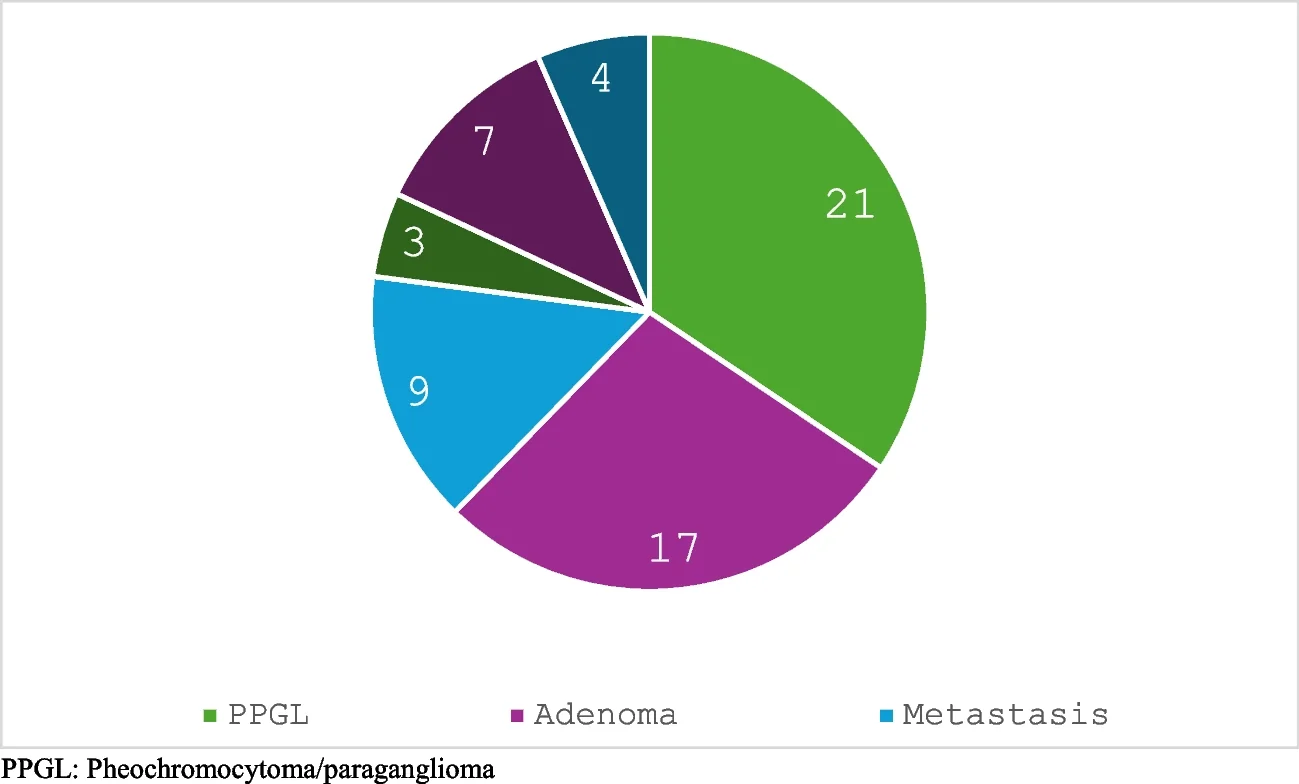
Histological diagnoses

Histological diagnoses included pheochromocytoma/paraganglioma (n = 21, 34%), adenoma (n = 17, 28%), metastasis (n = 9, 15%), primary malignancy (n = 3, 5%), other (n = 7, 11%), and myelolipoma (n = 4, 7%). Median lesion size was 4 cm overall, with larger lesions in open cases (6.5 cm) compared to minimally invasive (4 cm), consistent with selective use of open approaches for complex pathology (Fig. 2).

### Perioperative outcomes

**Fig. 3:**
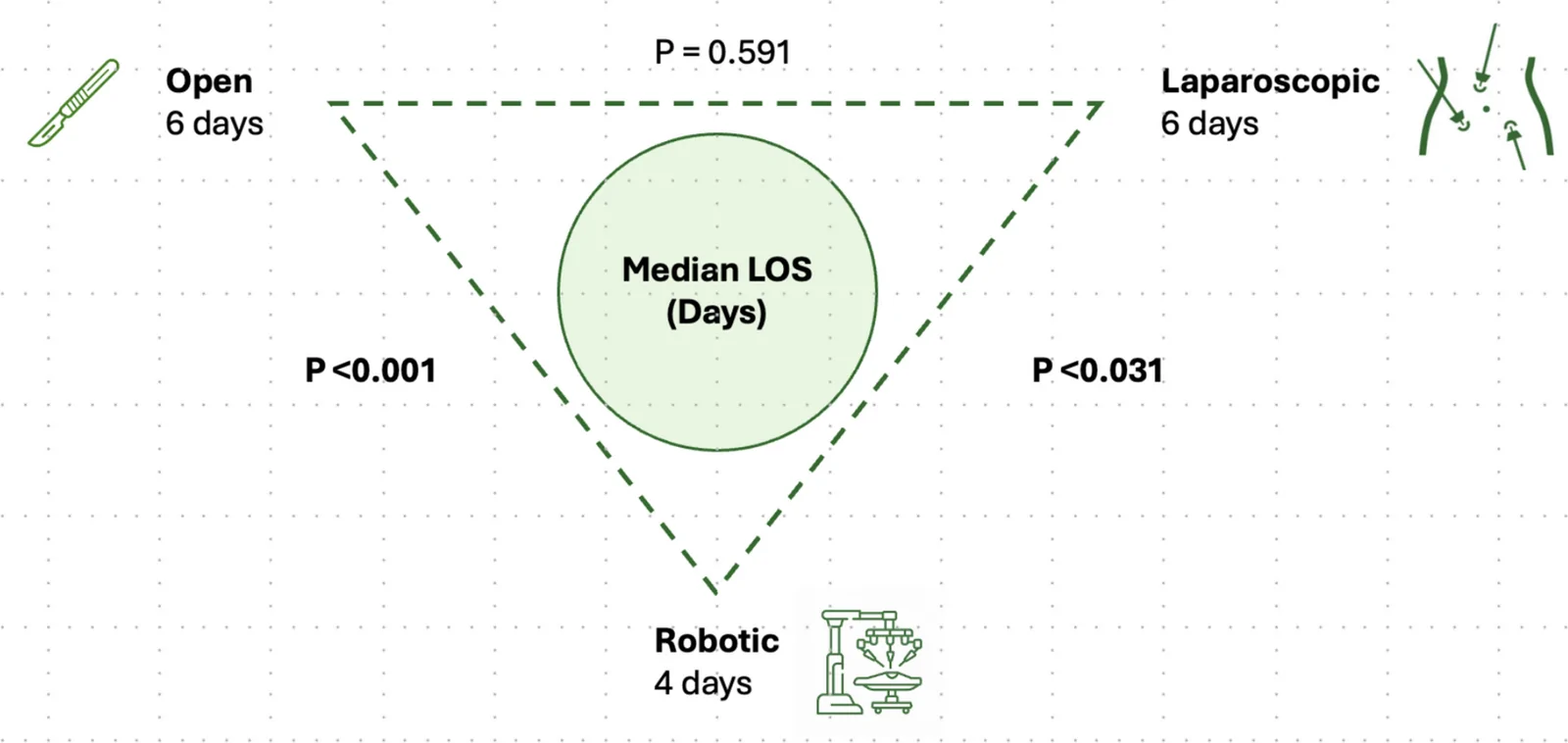
Length of stay by approach (p-values on bars)

Median LOS was 4 days for robotic cases, significantly shorter than 6 days for both open and laparoscopic (p < 0.001 vs. open; p < 0.031 vs. laparoscopic; p = 0.591 open vs. laparoscopic) (Fig. 3).

**Fig. 4:**
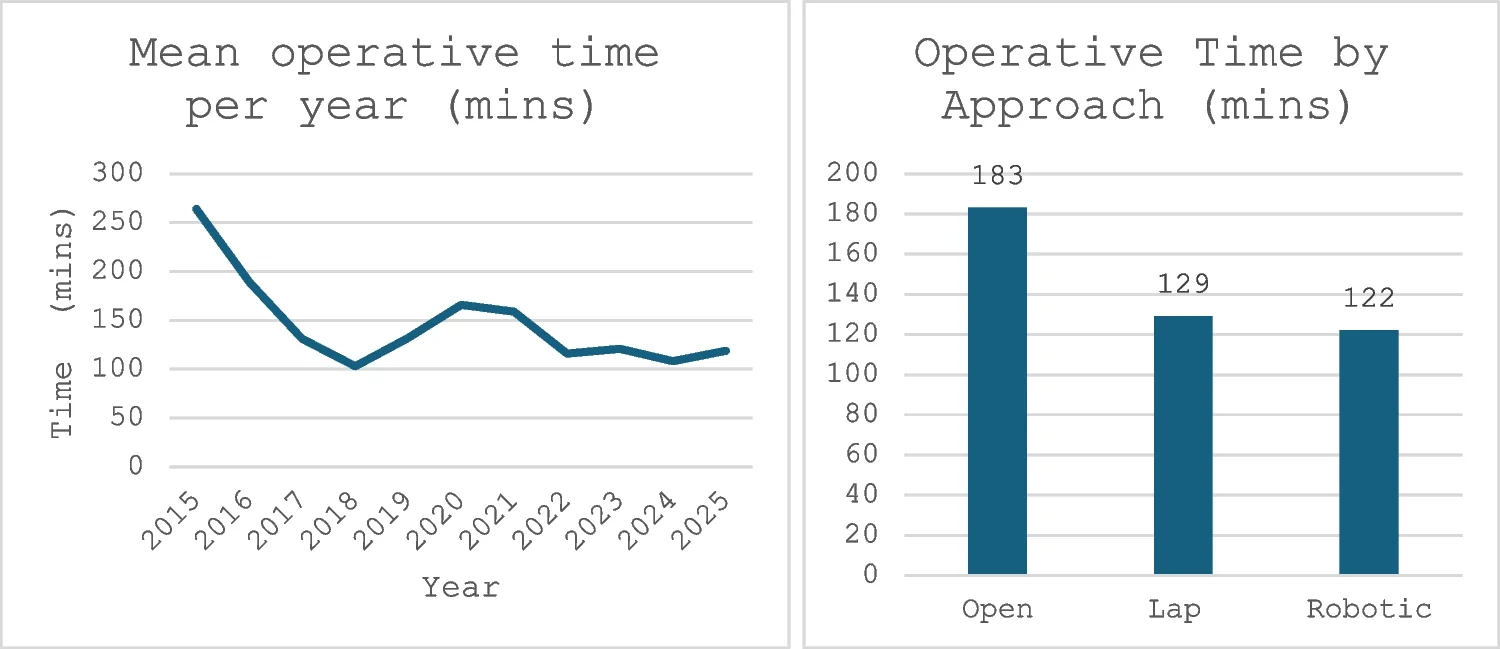
Operative time – annual trend and by approach

Mean operative time decreased annually from approximately 250 min in 2015 to 120 min in 2025, indicative of a learning curve. By approach, mean times were 183 min for open, 129 min for laparoscopic, and 122 min for robotic, with robotic significantly shorter than open (p < 0.006) and comparable to laparoscopic (Fig. 4).

**Fig. 5:**
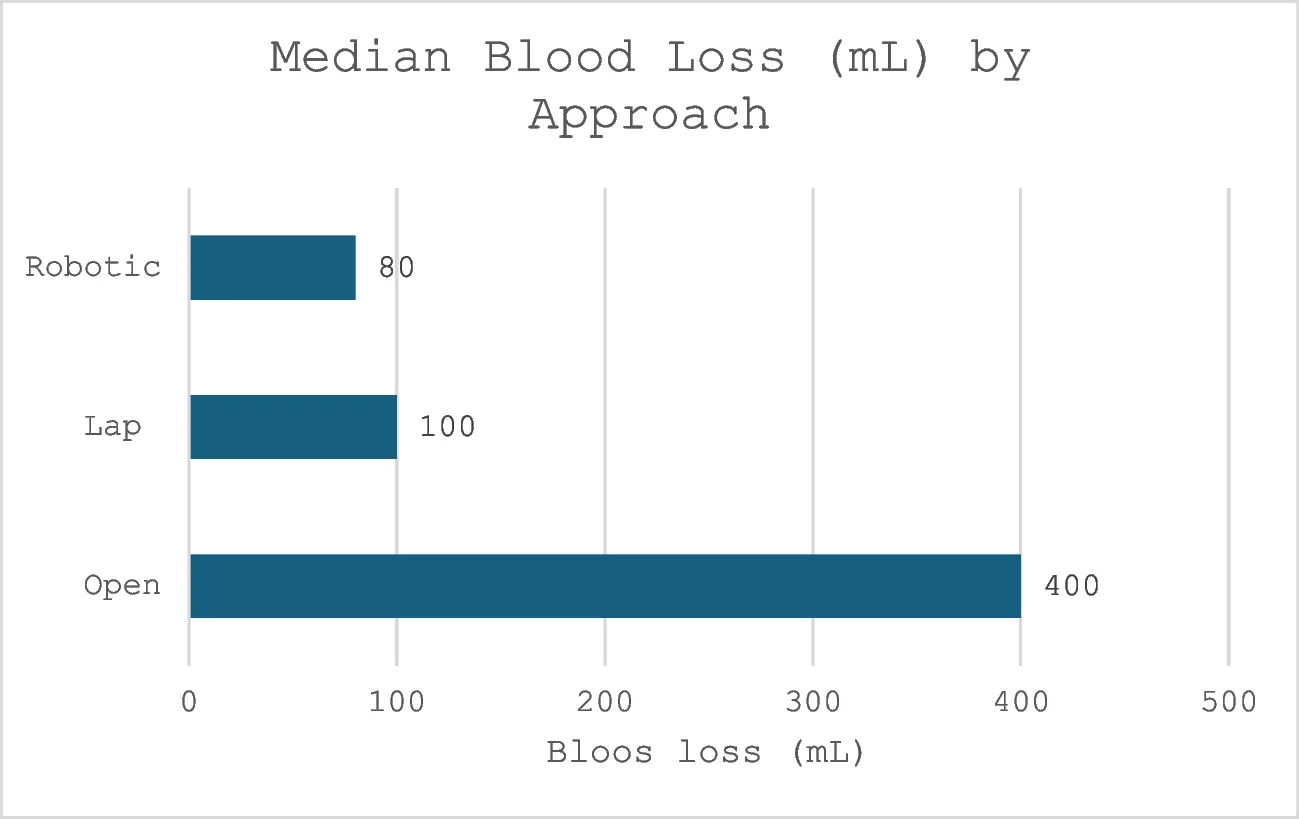
Estimated blood loss by approach

Median blood loss was 80 mL for robotic, 100 mL for laparoscopic, and 400 mL for open. Minimally invasive approaches showed significantly less blood loss than open (p < 0.001), with robotic outperforming laparoscopic (p < 0.05) (Fig. 5).

Overall 30-day Clavien-Dindo ≥ III complication rate was 4.9% (n = 3): one re-operation for bleeding (open case), one ICU admission for hypertensive crisis (laparoscopic pheochromocytoma), one readmission for adrenal insufficiency (robotic). No 30 or 90-day mortality reported.

## Discussion

This 10-year review at MMUH demonstrates a profound shift in adrenalectomy practice, from reliance on open surgery to predominant use of robotic techniques. Early years were characterized by low volumes and open approaches, likely due to established practices and limited access to robotic platforms. The introduction of robotic surgery post-2018 coincided with increased case volumes, driven by growing referrals, surgeon training, and institutional investment in the Da Vinci Xi system. By 2025, robotic adrenalectomy accounted for the majority of cases, aligning with global trends toward minimally invasive surgery for improved precision and ergonomics [4, 5].

Perioperative outcomes improved significantly with this transition but comparisons between groups are limited by baseline differences. However, taking this into account, there are clear benefits associated with the move to the robotic platform. Reduced LOS in robotic cases (4 days vs. 6 days) reflects faster recovery, consistent with literature showing benefits in pain control and mobilization [6]. Operative times decreased over time, suggesting a learning curve effect, with robotic times comparable to laparoscopic and shorter than open, without compromising safety [4]. Blood loss was markedly lower in robotic procedures, potentially due to enhanced visualization and instrument control, reducing transfusion needs and complications [4]. Larger lesions were appropriately managed openly, highlighting selective case allocation to optimize outcomes. Similar improvements in perioperative recovery have been observed in other single-centre retrospective series evaluating the transition from open to minimally invasive techniques [8].

Limitations include the retrospective design, small sample size (n = 61), and single-centre focus. The comparison between open, laparoscopic, and robotic approaches is limited by clear baseline differences: Open cases involved significantly larger and more complex lesions, surgical approach selection was era-dependent and case allocation was non-randomised. These factors substantially bias outcome comparisons. As a result, formal comparative effectiveness conclusions cannot be drawn. Long term outcomes (e.g., recurrence, endocrine function) were not fully captured, warranting future prospective studies.

Another limitation of this study’s ability to compare techniques is learning-curve bias. Robotic cases were performed predominantly after 2020, so improvements in operative time, LOS and blood loss may partly reflect accumulating surgeon and team experience rather than the robotic platform itself. This temporal confounding is inherent in single-centre technology-adoption series and cannot be excluded without contemporaneous controls.

Despite this, the data underscore the feasibility and benefits of robotic adoption in an Irish setting, where adrenal surgery volumes are modest compared to international high-volume centres [6].

## Conclusion

Over a decade, MMUH’s HPB service transitioned successfully to robotic adrenalectomy, accompanied by rising case volumes and substantial improvements in LOS, operative time, and blood loss. This evolution enhances patient care and supports continued investment in robotic technology for adrenal surgery.
